# Category Processing and the *human likeness dimension* of the Uncanny Valley Hypothesis: Eye-Tracking Data

**DOI:** 10.3389/fpsyg.2013.00108

**Published:** 2013-03-07

**Authors:** Marcus Cheetham, Ivana Pavlovic, Nicola Jordan, Pascal Suter, Lutz Jancke

**Affiliations:** ^1^Department of Neuropsychology, University of ZurichZurich, Switzerland

**Keywords:** categorization, sex differences, human likeness, uncanny valley hypothesis, eye movement

## Abstract

The Uncanny Valley Hypothesis (Mori, 1970) predicts that perceptual difficulty distinguishing between a humanlike object (e.g., lifelike prosthetic hand, mannequin) and its human counterpart evokes negative affect. Research has focused on affect, with inconsistent results, but little is known about how objects along the hypothesis’ dimension of human likeness (DHL) are actually perceived. This study used morph continua based on human and highly realistic computer-generated (avatar) faces to represent the DHL. Total number and dwell time of fixations to facial features were recorded while participants (*N* = 60) judged avatar versus human category membership of the faces in a forced choice categorization task. Fixation and dwell data confirmed the face feature hierarchy (eyes, nose, and mouth in this order of importance) across the DHL. There were no further findings for fixation. A change in the relative importance of these features was found for dwell time, with greater preferential processing of eyes and mouth of categorically ambiguous faces compared with unambiguous avatar faces. There were no significant differences between ambiguous and human faces. These findings applied for men and women, though women generally dwelled more on the eyes to the disadvantage of the nose. The mouth was unaffected by gender. In summary, the relative importance of facial features changed on the DHL’s non-human side as a function of categorization ambiguity. This change was indicated by dwell time only, suggesting greater depth of perceptual processing of the eyes and mouth of ambiguous faces compared with these features in unambiguous avatar faces.

## Introduction

**Figure 1:**
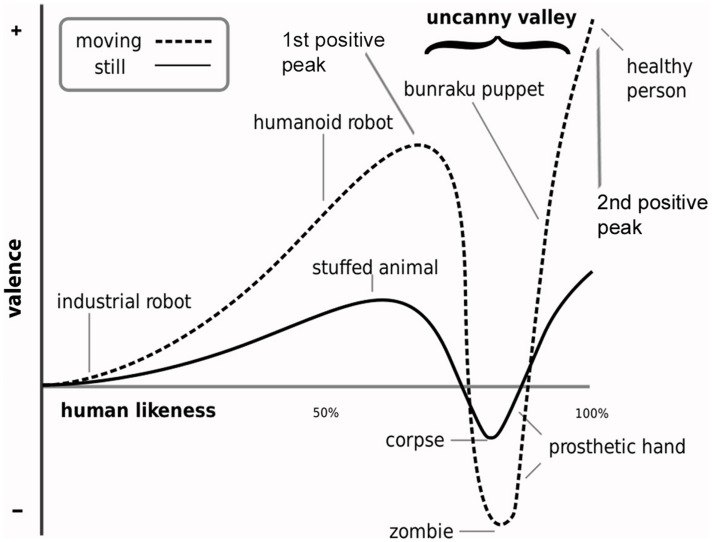
**Uncanny valley hypothesis**. The illustration shows the hypothesis’ proposed non-linear relationship between physical humanlike realism and affective experience. This relationship is generally positively valenced except at the degree of humanlike realism at which an object’s appearance and behavior is difficult to distinguish from that of the real human counterpart (i.e., at the first positive peak). This difficulty results in a sharp negative peak in the valence of affective experience that Mori describes as including feelings of eeriness and the uncanny (i.e., uncanny valley). This uncanny effect is thought to be stronger for objects in motion (illustration from MacDorman, 2005).

The Uncanny Valley Hypothesis (Mori, 1970) predicts that difficulty distinguishing between a humanlike object (e.g., robot, lifelike prosthetic hand, mannequin) and its natural human counterpart will evoke negatively valenced feelings and cognitions (for recent overviews, see, MacDorman et al., 2009a; Pollick, 2010). Mori described this negative state as characterized by feelings of unease and the uncanny (Figure 1). This prediction has been of concern to animators, video game designers and roboticists in their effort to ensure that the appearance of highly realistic humanlike characters influences subjective experience and behavior in the way that its design intended (e.g., Fabri et al., 2004; Minato et al., 2006; Walters et al., 2008; MacDorman et al., 2009a; Ho and MacDorman, 2010). Uncanny experience and the hypothesis’ valence dimension have therefore received much research attention. But this research has produced inconsistent results (e.g., Hanson, 2006; MacDorman, 2006; Tinwell and Grimshaw, 2009; Tinwell et al., 2010). One likely reason for this inconsistency is the uncertainty surrounding the vague terminology used to describe the hypothesis’ valence dimension (e.g., Bartneck et al., 2007; Seyama and Nagayama, 2007; MacDorman et al., 2009a; Dill et al., 2012). Another reason might be related to the fact that the hypothesis’ *dimension of human likeness* (*DHL*), defined in terms of a linear change in the degree of physical humanlike similarity, is not subjectively perceived as a simple linear change in humanlike similarity (Cheetham et al., 2011; Cheetham and Jancke, 2013). Given the aim of uncanny research to understand subjective experience of nonhuman characters in terms of human likeness, this recent evidence suggests that a better understanding of how human likeness is really perceived is needed.

One approach to investigating perception along the DHL is to record eye movement (Just and Carpenter, 1980; Goldberg and Wichansky, 2003). Eye movements orient foveal vision to perceptual details that are critical for encoding and perceiving objects (Yarbus, 1967; Loftus and Mackworth, 1978; Desimone and Duncan, 1995; Egeth and Yantis, 1997(for reviews, see Rayner, 1998; Henderson, 2003, 2007). Investigation of eye movement has contributed to our understanding of face perception (e.g., Walker-Smith et al., 1977; Cook, 1978; Schyns et al., 2002; Pearson et al., 2003; Stacey et al., 2005). These studies show that measures of eye movement such as the number of fixations and dwell time (i.e., the cumulative duration of fixations) over a particular area of interest are sensitive to bottom-up effects of stimulus information and to top-down task requirements (e.g., Robinson, 1964; Yarbus, 1967; Posner, 1980).

The Uncanny Valley Hypothesis essentially describes a situation in which individuals engage in the task of implicitly or explicitly assigning perceptually similar humanlike objects to a non-human or human category. An implicit assumption of the hypothesis is that this task can vary in difficulty along the DHL. This was confirmed by Cheetham et al. (2011). They used human and highly realistic avatar faces to generate morph continua to represent the DHL and presented the morphs of these continua in a *two-alternative forced choice categorization task*. This task required participants to assign membership of each morphed face to either the human or avatar category. Their measures of response accuracy and response time confirmed that task difficulty increases sharply at the category boundary, that is, at the point of greatest categorization ambiguity. Similar findings have been reported for continua based on other stimuli (e.g., Campbell et al., 1997). Consistent with Mori’s informal description of the hypothesis, this boundary should therefore mark the point along the DHL at which perceptual difficulty in extracting the visual evidence required to inform the category decision is most likely to evoke uncanny experience. The relationship between perceptual difficulty and uncanny experience has yet to be tested.

Greater task difficulty can also be indicated by a longer duration of fixations, that is, dwell time (Buswell, 1935). Dwell time is thought to reflect the demands of actively processing a fixated region of visual interest (for process monitoring models see, Rayner, 1987; Rayner and Fischer, 1987) and of the degree of in-depth processing of task-relevant information (Duncan and Humphreys, 1989; Remington and Folk, 2001; Becker, 2011). For example, longer dwell time can reflect greater processing demands when discriminating between similar stimuli (Shen et al., 2003; Becker, 2011) and when matching observed visual stimulus information to internal representations (Goldberg and Kotval, 1999). Uncertainty in perceptual decision making entails enhanced recruitment of attentional resources for the accumulation of sensory evidence (Heekeren et al., 2008). Longer dwell time might therefore be expected when an individual encounters difficulty extracting perceptual information from a categorically ambiguous stimulus and matching this information to representations of the non-human or human category (Fitts et al., 1950; Barton et al., 2006). Assuming that Mori’s hypothesis is correct, the cognitive demands placed on processing perceptual information should be greater at the category boundary of the DHL compared with the other regions of the DHL where there is little or no categorization ambiguity. Based on the preceding considerations, it would be consistent with Mori’s hypothesis that differences in perceptual processing demands along the DHL are indicated by differences in dwell time.

The relative importance of particular visual features such as the eyes, nose, and mouth is not addressed in the Uncanny Valley Hypothesis, although these features have received attention in uncanny-related research (e.g., Seyama and Nagayama, 2007; MacDorman et al., 2009a; Looser and Wheatley, 2010). Certain facial features and particularly the eyes do generally draw more overt attention in various tasks (Yarbus, 1967; Fisher and Cox, 1975; Haith et al., 1977; Walker-Smith et al., 1977; Janik et al., 1978; Langdell, 1978; Althoff and Cohen, 1999; Minut et al., 2000; Henderson et al., 2005; Schwarzer et al., 2005). Evidence of a *facial feature hierarchy* pertaining to the relative importance of the eyes, nose, and mouth (and of other features) is long established (Ellis et al., 1979; Fraser and Parker, 1986). But a central question affecting both design considerations and the cognitive processing of such realistic humanlike characters is whether the relative importance of these facial features is modulated by categorization ambiguity. In other words, does the relative importance of these facial features change as a function of the difficulty of perceptual decision making at different points along the DHL? Uncanny-related research shows that the relative *realism* of the eyes can influence the experience of negative affect (Seyama and Nagayama, 2007; MacDorman et al., 2009a), and a recent study suggests that the eyes and mouth contain more diagnostic information for determining whether human and humanlike faces are animate (Looser and Wheatley, 2010). These findings mean that certain facial features might be preferentially fixated in order to determine category membership when sensory evidence is unclear. This would be indicated by a change between categorically unambiguous and ambiguous faces in the relative proportion of fixations to the eyes, nose, and mouth.

An important consideration is that women show a general processing advantage for perceptual details in face detection and facial identity discrimination tasks, especially under more demanding processing conditions (McBain et al., 2009; see also Lewin and Herlitz, 2002; Rehnman and Herlitz, 2007). This advantage is well investigated for face recognition (for a review, see Herlitz and Rehnman, 2008) and facial expression recognition (Kirouac and Doré, 1985; Nowicki and Hartigan, 1988; Thayer and Johnsen, 2000; Hall and Matsumoto, 2004; Montagne et al., 2005; Scholten et al., 2005; Biele and Grabowska, 2006). These studies show that women process faces faster and more accurately than do men. The female advantage in expression recognition is associated with greater female attention to the eyes, indicated by longer eye-related dwell time and larger fixation number (Hall et al., 2010). Similar findings have been found in other tasks and with other stimuli (Miyahira et al., 2000a,b).

The aim of this investigation was to examine whether there are differences in the way visual attention is overtly oriented to the eyes, nose, and mouth of categorically unambiguous and ambiguous faces along the DHL. The DHL was represented using morph continua (e.g., Hanson, 2006; Seyama and Nagayama, 2007; Ho et al., 2008), the advantage of which is that they allow careful experimental control of differences in humanlike appearance and the exclusion of confounding perceptual dimensions (for a critical overview of the use of morph continua in uncanny research, see Cheetham and Jancke, 2013). The morph continua were generated from avatar and human parent faces and the morphs presented in a forced choice categorization task. Eye measures of fixation number and dwell time were collected to determine, firstly, the presence of a general face feature hierarchy for the eyes, nose, and mouth. Second, we examined whether the relative importance of these features changes between categorically unambiguous avatar and human faces compared with categorically ambiguous faces at the category boundary of the DHL. The categorically unambiguous faces and the peak in categorization ambiguity were determined on the basis of the categorization responses and response times (RT) in the categorization task. Based on the preceding considerations, we predicted that greater categorization ambiguity would be reflected in a shift in the relative importance of facial features in terms of dwell time but not necessarily in terms of the actual location of fixations. Third, we examined whether there are differences between men and women in categorization responses and response latencies and in our measures of eye movement. Based on the available literature, we anticipated that women would generally show shorter response latencies than men and explored the possibility that the eyes are generally more important for women than for men when processing faces along the DHL.

## Materials and Methods

### Participants

All volunteers were healthy students (*N* = 60, 31 females, 29 males; aged 18–32, mean 23 years) of the University of Zurich, with no record of neurological or psychiatric illness and no current medication. All participants had normal or corrected-to-normal visual acuity, were native or fluent speakers of Swiss or Standard German, and consistently right-handed (Annett, 1970). All participants reported having no active design or gaming experience with computer-generated characters in video games, virtual role-playing games, second life and other virtual reality environments. One female participant was omitted from analyses because she did not show a logistic component in the response function of her data (see section Logistic Function of Categorization Responses). Heavy track loss, that is, failure by the eye-tracking system to detect the pupil (mostly in this study because of excessive blinking) resulted in there being no fixation data in over 20% of trails in another female and in five male participants. The data of these participants was excluded from further analysis. Written informed consent was obtained before participation according to the guidelines of the Declaration of Helsinki. Each volunteer received 15 Swiss Francs for participation. The study and all procedures and consent forms were approved by the Ethics Committee of the University of Zurich.

### Materials and stimuli

#### Stimuli

Ten morph continua were generated using Morpher 3.3 software (Zealsoft, Inc., Eden Prairie, MN, USA) to represent the DHL. The parent images of the continua were natural human and avatar faces. Each face was male, indistinctive, presented with full frontal view, direct gaze, neutral expression and no other salient features (e.g., facial hair, jewelry). The avatars were generated using the avatar modeling software Poser 7 (Smith Micro Software)^1^. The avatars’ facial geometry and texture (configural cues, skin tone) were modeled to closely match the human counterpart. The parent images were then edited using Adobe Photoshop CS3. The external features of each parent image were masked with a generic elliptic form and black background, and contrast levels, overall brightness of the parent images of each continuum were adjusted to match. Each morph continuum comprised 15 different morphed images, each morph separated by an increment of 6.66% in physical difference. A pilot study of *N* = 20 participants who were not recruited for the present study was conducted using the morph images of each morph continua in a forced choice categorization task. These images were numbered 1 (avatar end) to 15 (human end). The purpose of the pilot study was to verify that the morph position along the continua of the category boundary and the shape of the response function of each continuum was consistent across all continua before performing the present eye-tracking study. This was confirmed. The task and analyses for the pilot study were the same as described in the following sections for the present study. Based on the pilot study, the morph images 3–15 (i.e., the 13 most human-like morphs) of each morph continuum were used in the present study and re-labeled 1 (avatar end) to 13 (human end; Figure 3). This was done to achieve a better balance in the number of morph images either side the category boundary. At a viewing distance of 62 cm, the stimuli (400 × 500 pixels) subtended a visual angle of 11° × 14°. This is approximately equivalent to viewing a real face from a normal distance during conversation of 90–100 cm (Hall, 1966; Henderson et al., 2005).

### Design and procedure

The participants were examined individually, each examination lasting approximately 20 min in total. The participant received general instructions before beginning each experimental phase (i.e., calibration of eye-tracker, practice pre-test, and main test). The participant was seated in a chair with a chin rest and head band to restrain head movement. An I-View SMI dark pupil remote eye-tracker system (SensoMotoric Instruments, Gmbh) with sampling frequency at 50 Hz and up to 0.5° accuracy was used to record horizontal and vertical eye positions. A 13-point calibration procedure was applied at the beginning of the experiment and drift correction was performed automatically. After the calibration procedure, the participant read written instructions on the monitor before commencement of the forced choice categorization task. This began with a practice pre-test of five trials using stimuli drawn from an unused continuum. Comprehension of the task and correct use of the response buttons was ensured. The participant then pressed the response box to initiate trial presentation for the main test.

Each trial started with a blank screen for 2200 ms to allow rest and blinks followed by a fixation point for 800 ms (prepare signal) and then a stimulus. Participants were instructed to fixate on the point until the stimulus appeared, to view the stimulus freely and to identify the stimulus quickly and accurately as either an avatar or human by pressing one of two response keys. The stimulus disappeared from view following button press or after the maximum viewing duration of 3000 ms. Participants performed 130 trials, with each morph of each continuum being presented once, individually, in the center of the monitor and in random order.

The task was conducted in a sound attenuated and light-dimmed room, and stimuli were presented on a LCD monitor (1280 × 1024 resolution, 60 Hz refresh rate, at eye-to-monitor viewing distance of 62 cm), using Presentation^®^ software (Version 14.1)^2^.

### Analyses

**Figure 2:**
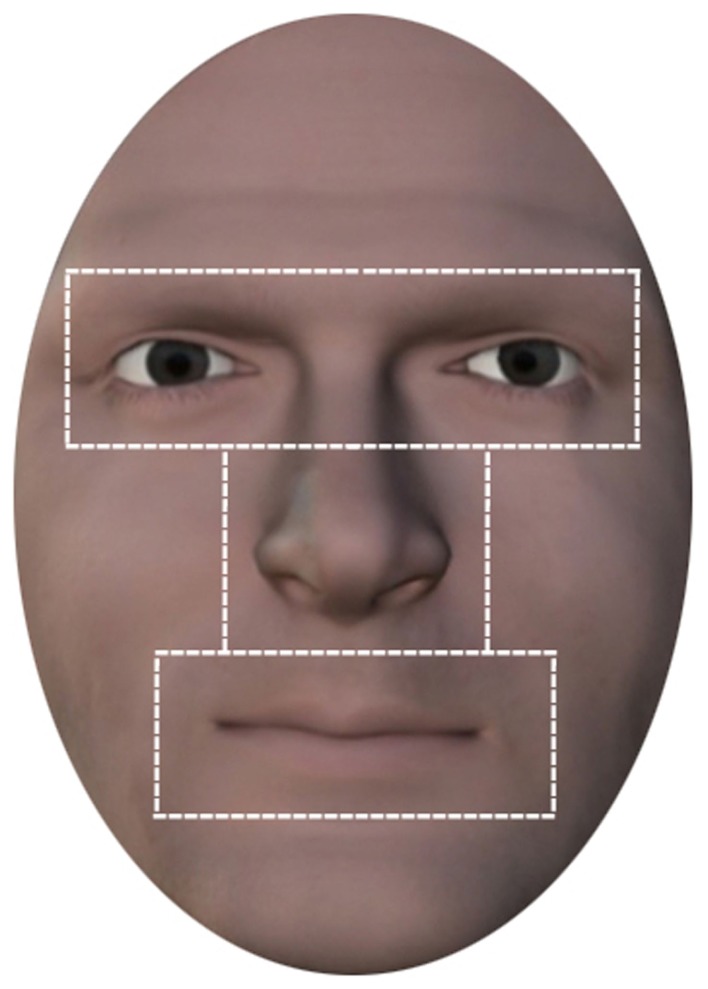
**Areas of interest**. Illustration of internal region of the face comprising the areas of interest for the facial features eyes, nose, mouth.

Eye movement data from stimulus onset until participant response was analyzed. Be-Gaze software Version 2.5 (SensoMotoric Instruments, Gmbh) was used to pre-process the data. A fixation was defined as consecutive eye gaze positions within an area of 1° for a period of 100 ms or more. Blinks and any fixations that fell outside the masked face region were discarded from data analysis. Four areas of interest (AOI) were assigned: the internal face area as a whole and the individually defined areas of the eyes (including the eyebrows), nose, and mouth, as shown in Figure 2. Total fixation number and cumulative total fixation duration for each AOI was computed. The same AOI were applied to all stimuli to ensure consistency in the definition of boundaries between facial features and size of AOI. Given the different response latencies, values were normalized to represent the “proportion of the total number of fixations” (referred to in the following as “fixations”) and the “proportion of the total fixation duration” (referred to in the following as “dwell time”) within each AOI and entered into statistical analyses as dependent variables. For all analyses, only data was included for trials in which responses were made and before stimulus duration elapsed. All data analyses were performed using SPSS version 17.0 (SPSS, Inc., Chicago, IL, USA).

## Results

### Forced choice categorization

#### Forced choice – categorization responses

The slope of the categorization response function was used to summarize the avatar-human categorization response data. The function was determined by fitting logistic function models to the data of each participant across continua. Informal inspection of each participant’s fitted regression curve indicated for each participant a response function with a sigmoid shape indicative of a category boundary, with the exception of one participant who following the analysis of logistic function (see section Logistic Function of Categorization Responses) was excluded from subsequent analyses. Parameter estimates were derived from the model and entered in the analyses of the logistic function of categorization responses and of the category boundary.

**Figure 3:**
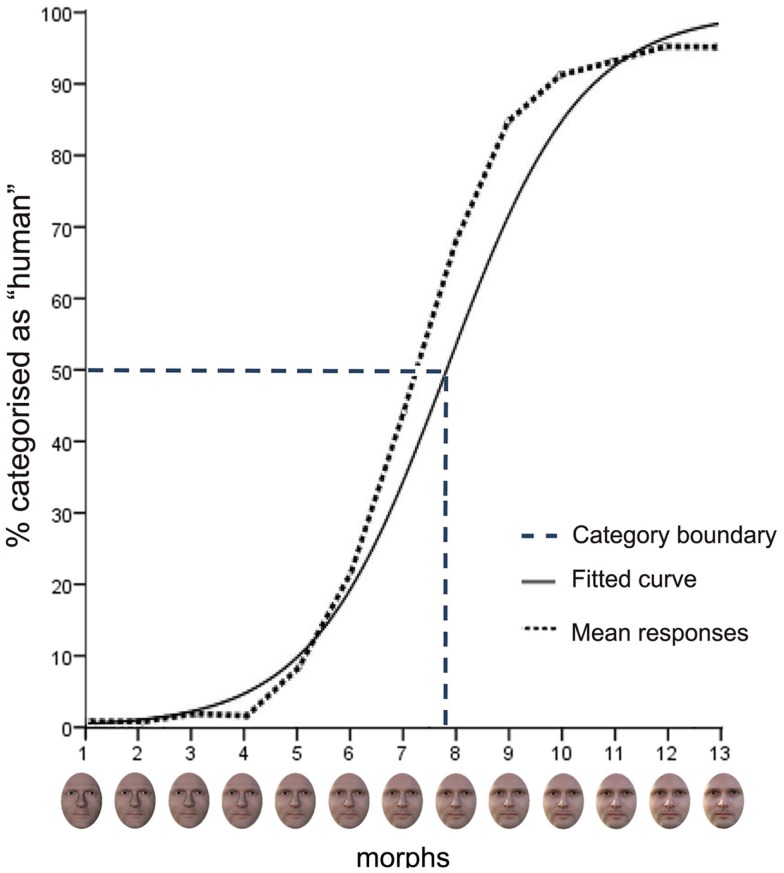
**Mean responses in the categorization task**. The mean aggregated response (dotted line), shown in terms of the percentage of “human” responses, and the mean logistic curve (continuous line) across all continua and participants are displayed. The mean category boundary (dashed line) indicates the morph position along the continua at which there is maximum uncertainty of 50% in categorization judgments. The bottom panel of the figure shows an example of a morph continuum.

Figure 3 shows the mean aggregated response data for the 10 continua across male and female participants and, for purposes of illustration, the fitted response function computed on the basis of the grand mean of these continua. The sigmoid-shaped curve of the fitted response function shows a lower and upper asymptote of avatar and human categorization responses that nears 100% for avatars and 95% for humans, respectively.

#### Logistic function of categorization responses

The derived parameter estimates for the logistic function of data averaged across the 10 continua for each participant were tested against zero in a one-sample *t*-test. The result showed a highly significant logistic component, *t_58_* = 20.19, *p* > 0.001, that captures a sigmoid-shaped function consistent with a category boundary (Harnad, 1987; Figure 3).

#### Category boundary

The estimates for the β0 and β1 parameters of each participant across continua were used to compute the *category boundary value* [i.e., *y* = 0.5: −ln(β0)/ln(β1)]. This value indicates the morph position along the continua that corresponds with the ordinate midpoint between the lower and upper asymptotes, that is, the point of maximum uncertainty of 50% in categorization judgments. Across continua, the mean category boundary value (*M* = 7.81, SD = 0.83) corresponds with face morph position 8 (Figure 3). An independent *t*-test showed that the mean category boundary value for male participants (*M* = 8.23, SD = 0.93) was not significantly different from that for female participants (*M* = 7.49, SD = 0.68).

#### Categorization response as a function of morph position

We tested for expected differences in the categorization responses as a function of morph position (i.e., 1–13) and examined potential differences in categorization responses between the continua and male and female participants. A repeated measures of analysis of variance (RM-ANOVA) was performed on the dependent variable “response” (for avatar-human categorizations) of each participant with the “morph position” (13 levels) across the 10 continua. “Sex” was entered in analysis as between subject variable. Greenhouse–Geisser adjustment was applied to correct the degrees of freedom for violation of the sphericity assumption (and applied as appropriate in subsequent analyses). The analysis showed a highly significant effect for morph position [*F*(4.39, 259.23) = 765.48, *p* < 0.001].

#### Forced choice – categorization response times

**Figure 4:**
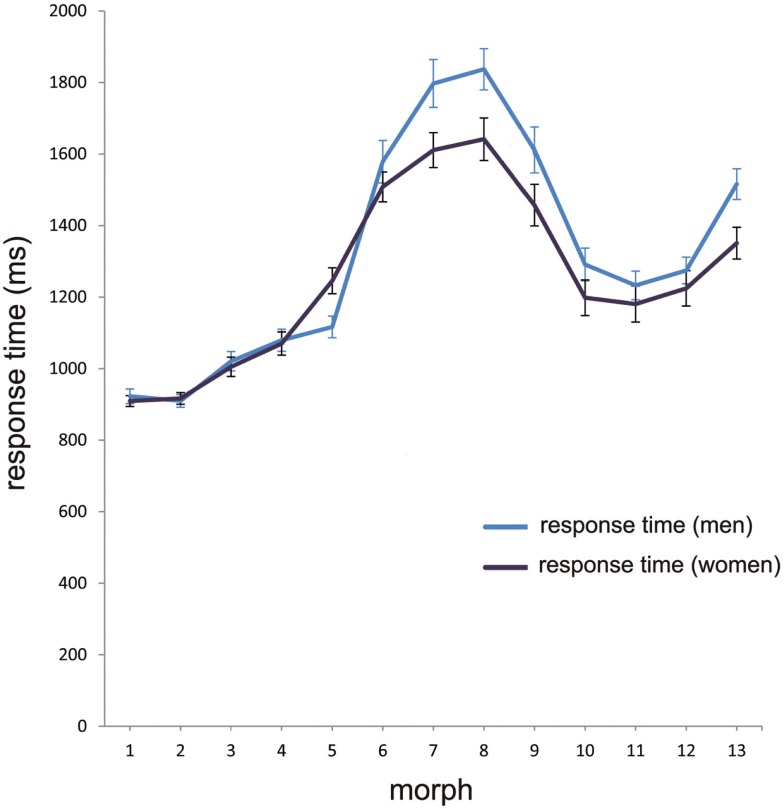
**Mean RT in the categorization task**. The mean aggregated response time for men (blue line) and women (black line) at the different morph positions 1–13, with error bars as standard errors (*N* = 59). Note that the longest response latencies for men and women at morph 8 correspond with the category boundary shown in Figure 2.

Differences in category decision difficulty, as indicated by the slope of the categorization response function, are likely to be reflected in different response latencies for the morphs of the continua. To gain an overall picture of the RT at different morph positions, a one-way RM-ANOVA with *morph position* (13 levels) and RT as the dependent variable was conducted. “Sex” was entered in analysis as between subject variable. The analysis showed a highly significant effect for morph position [*F*(4.28, 252.3) = 168.2, *p* < 0.001], and for sex, *F*(4.28, 252.3) = 4.54, *p* = 0.001 (Figure 4).

The longest response latency should correspond with the position of the category boundary. For the whole sample, the category boundary is morph 8. To characterize the effect more clearly, the mean RT values at morph 8 were compared with the mean RT values at all other morph positions. A one-way RM-ANOVA analysis with *morph position morph 8* versus *all other morphs* and RT as dependent variable and sex as between subject variable showed that RT at morph 8 (*M* = 1.73, SD = 0.33) differed highly significantly from RT for the other morph positions (*M* = 1.25, SD = 0.16), *F*(1, 58) = 158.93, *p* < 0.001. There was no effect for sex.

Given the interest in asymmetry in performance for within-category avatar and human faces, we compared mean RT for morph positions 1–4 with mean RT for the morph positions 10–13. These morph positions were unambiguously assigned to one or other category with a decision certainty of in excess of 90%; a much less conservative criterion is often used in CP research (e.g., 66% as in Ectoff and Magee, 1992; Beale and Keil, 1995). For this, a one-way RM-ANOVA was conducted using the factor *morph position* (two levels: 1–4, 10–13) and RT as dependent variable. This indicated that mean RT for avatars at morph positions 1–4 (*M* = 1.02, SD = 0.12) was significantly shorter than that for the human faces at morph positions 10–13 (*M* = 1.33, SD = 0.24), *F*(1, 58) = 135.29, *p* < 0.001. There was also a gender effect, such that males were slower to humans faces (*M* = 1.38, SD = 0.21) than females (*M* = 1.28, SD = 0.26), *F*(1, 58) = 5.14, *p* = 0.027 (Figure 4). There was no difference in RT between man and women for faces of the avatar category.

### Eye movement

#### Internal face area, and dwell time

To determine the relative importance of the internal face area comprising the eyes, nose, and mouth as a whole, a one-way RM-ANOVA was conducted using the factor *morph position* (13 levels: 1–13) and dwell as dependent variable. Sex was entered in the analysis as between subject variable. No significant differences were found.

#### Eyes, nose, mouth, and dwell time

**Figure 5:**
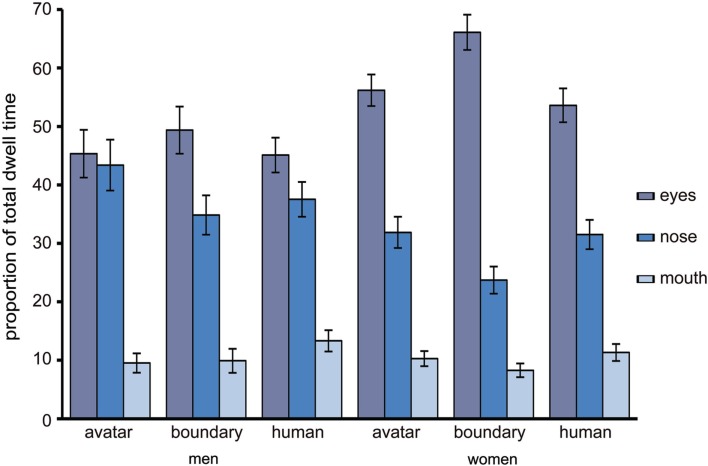
**The proportion of total fixation duration on the eyes, nose, and mouth**. The relative proportion of total dwell time to the eyes, nose, and mouth for the avatar and human categories and for the peak in uncertainty at the category boundary, with error bars as standard errors (*N* = 53).

To examine the relative role of eyes, nose, and mouth for the *DHL regions* avatar category, human category and for the morph of peak uncertainty (i.e., morph position 8) during category decision making, a two-way RM-ANOVA was conducted with the factors *DHL region* (3 levels: 1–4, 8, 10–13) and feature (three levels: eyes, nose, mouth) and dwell as dependent variable. “Sex” was entered in analysis as between subject variable. The analysis showed a general difference between men and women in the relative importance of the eyes, nose, and mouth, *F*(68.1, 1.36) = 4.47, *p* < 0.027. The pre-planned contrasts show that there were generally no significant differences for the mouth between men and women, while the eyes were more salient relative to the nose for women (*M*_eyes_ = 58.55, SE_eyes_ = 3.39; *M*_nose_ = 29.55, SE_nose_ = 2.95) than for men (*M*_eyes_ = 48.78, SE_eyes_ = 4.36; *M*_nose_ = 33.38, SE_nose_ = 3.83), *F*(1, 50) = 5.26, *p* < 0.026 (Figure 5).

The analysis showed also a significant interaction effect for ENM × DHL region [*F*(2.65, 132.46) = 4.24, *p* = 0.009] such that the relative salience of the eyes, nose, and mouth varies as a function of DHL region. At the peak compared with the avatar category, pre-planned contrasts show a significant increase in sampling of the eyes (*M*_peaks_ = 57.56, SE = 3.66; *M*_avatar_ = 50.34, SE = 3.42) compared with the nose (*M*_peak_ = 29.38, SE = 2.31; *M*_avatar_ = 37.84, SE = 3.16), *F*(1, 51) = 7.78, *p* < 0.007, and a significant decrease in sampling of nose (*M*_peak_ = 29.38, SE = 2.31; *M*_avatar_ = 37.84, SE = 3.16) compared with the mouth (*M*_peak_ = 8.43, SE = 1.17; *M*_avatar_ = 10.62, SE = 1.45), *F*(1, 51) = 0.78, *p* < 0.033. At the human compared with the avatar category, there was a significant decrease in sampling of the nose (*M*_human_ = 34.65, SE = 2.74; *M*_avatar_ = 37.84, SE = 3.62) relative to the mouth (*M*_human_ = 11.62, SE = 1.43; *M*_avatar_ = 10.07, SE = 1.45), *F*(1, 51) = 6.96, *p* < 0.011, but no significant effect for eyes.

#### Internal face area, and fixation number

A one-way RM-ANOVA was conducted using the factor *morph position* (13 levels: 1–13) and dwell as dependent variable, and with sex as between subject variable. There were no effects.

#### Eyes, nose, mouth, and fixation number

**Figure 6:**
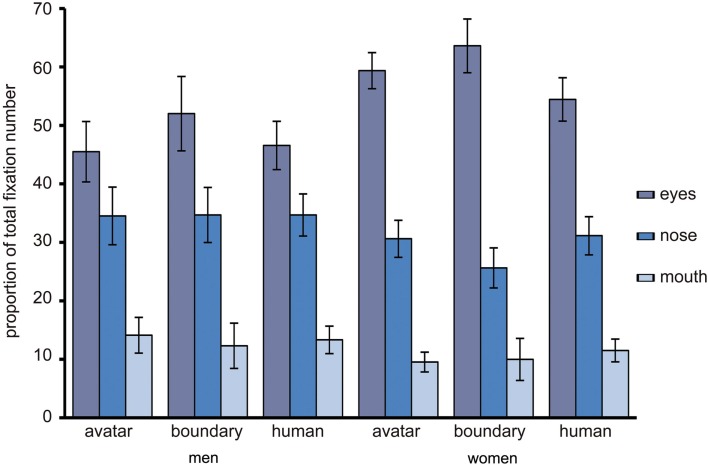
**The proportion of total fixation number to the eyes, nose, and mouth**. The relative proportion of total fixation number to the eyes, nose, and mouth for the avatar and human categories and for the peak in uncertainty at the category boundary, with error bars as standard errors (*N* = 53).

To examine the relative role of eyes, nose, and mouth for the three DHL regions, as in the preceding analysis, a two-way RM-ANOVA was conducted with the factors *DHL region* (three levels: 1–4, 8, 10–13) and feature (three levels: eyes, nose, mouth) and fixations as dependent variable. “Sex” was entered in analysis as between subject variable. The analysis showed a highly similar general pattern for fixation as for dwell. There was a main effect for facial feature such that the eyes, nose, and mouth are differently important, *F*(1.53, 76.53) = 58.91, *p* < 0.001. The pre-planned contrasts show that the eyes were more salient relative to the nose (*M*_eyes_ = 54.01, SE_eyes_ = 3.23; *M*_nose_ = 31.67, SE_nose_ = 2.71) [*F*(1, 51) = 116.61, *p* < 0.001] and mouth (*M*_mouth_ = 11.55, SE_mouth_ = 1.93) *F*(1, 51) = 21.63, *p* < 0.001 and the nose more salient than the mouth, *F*(1, 51) = 53.12, *p* < 0.001 (Figure 6). But there was no significant effect for DHL region, though this did approach significance, *F*(148.9, 74.44) = 3.27, *p* = 0.058. There were no other significant effects for fixations.

## Discussion

The unique feature of uncanny research is the interest in understanding the impact on subjective experience and behavior of uncertainty in distinguishing highly realistic non-human objects (or specific perceptual attributes of these) from their human counterpart (Ramey, 2005; MacDorman et al., 2009b). In terms of categorization behavior, ambiguity in assigning category membership should be greatest at the category boundary (e.g., Campbell et al., 1997). For our avatar-human continua, a category boundary was indicated by the sigmoid shape of the response function (Harnad, 1987). This shape reflects a monotonic increase in categorization accuracy (i.e., the proportion of decision responses in favor of the avatar or human category) and a corresponding monotonic decrease in response latency as a function of morph distance from the category boundary. The data thus indicate that the category boundary marks the point of greatest cognitive conflict between the two competing categorization response tendencies.

Overt orientation of visual attention to the eyes, nose, and mouth was therefore compared between faces at the category boundary and faces that were unambiguously assigned to the avatar or human category. The data for the number of fixations showed no significant differences in this comparison, indicating that category ambiguity has no specific effect on the targeting of fixations to these facial features. But this data did confirm that the relative importance of features in the *face feature hierarchy* (e.g., Walker-Smith et al., 1977; Althoff and Cohen, 1999) is similar for faces of variously humanlike appearance along the DHL, the eyes being especially important. The latter might be expected given the high human sensitivity to eye information (Itier et al., 2007; Itier and Batty, 2009) and the diagnostic value of the eye region for various face processing tasks (e.g., Althoff and Cohen, 1999; Hall et al., 2010). This eye dominance might also reflect the influence of an automatic gaze strategy that entails positioning fixations near a center of gravity (Bindemann et al., 2009). This center of gravity is biased toward a position between the eyes and nose (e.g., Deaner and Platt, 2003; Grosbras et al., 2005; Tyler and Chen, 2006; Hsiao and Cottrell, 2008; Saether et al., 2009; van Belle et al., 2010). It is possible that the presentation of full frontal faces in this study facilitated the targeting of the eyes (Saether et al., 2009). On the other hand, fixations to the eye region might have been advantageous in our categorization task because this point of regard might best serve rapid visual inspection of the faces, with the eyes being processed foveally and the nose and other areas parafoveally. Compared with our full frontal face stimuli, a change in the relative viewpoint of the observer might alter the fixation patterns generally. This is likely because head (and body) orientation can influence the direction of the viewer’s attention (Hietanen, 1999, 2002; Langton, 2000; Pomianowska et al., 2011). The impact of different viewpoints on eye movement patterns when processing categorically unambiguous and ambiguous faces along the DHL is not known.

The data for dwell time revealed a different picture. Categorization ambiguity influenced the relative amount of dwell time spent extracting visual information from the eye, nose, and mouth before making a category decision. This effect was associated with the non-human side of the DHL, with a relative shift away from the nose region in avatars to the regions of the eyes and mouth of faces at the category boundary. A similar effect almost reached significance for the comparison of the human versus ambiguous faces. The eye movement data thus suggest that avatars are processed differently than are ambiguous faces. The behavioral data support this by showing that category assignment of avatar faces is also much faster in comparison with the assignment of human faces. This replicates Cheetham et al.’s (2011) finding, using a much larger participant sample in the present study. Given that our participants’ perceptual and categorization experience with novel non-human but highly humanlike faces generally and especially with those presented in this study does not compare to their everyday expertise with human faces (Diamond and Carey, 1977; Tanaka and Curran, 2001; see, Ramey, 2005), one might have expected that the assignment of faces to the human category would have been easier than to the avatar category.

One possible explanation for the strong difference in RT for the avatar compared with the human category decisions is that this decision is influenced by a strategy that involves the detection of perceptual information that is diagnostic of the non-human category, that is, of the category of which the human participant is *not* a member (see the *race-feature hypothesis*, Levin, 2000). This would mean that classification decisions are be based on establishing the presence or absence of avatar-specifying perceptual information, with faces being coded and categorized in terms of “avatar or not avatar” rather than as “avatar or human.” Assuming that detecting the presence rather than the absence of perceptual information is cognitively less demanding, this classification strategy would result in a classification advantage in RT for avatar faces. This *avatar-feature hypothesis* thus suggests that there might be preferential processing of avatar-specifying information during explicit categorization of categorically unambiguous avatars.

The question is what perceptual information might be diagnostic for avatar faces during categorization. One possibility, given the task’s context of processing novel avatar faces and faces of the familiar human category, is that perceptual information indicating novelty could in itself serve as a readily identifiable primitive feature of members of the avatar category (cf. Levin, 2000). Certain properties of an avatar’s face such as the general shading of the smoothed skin texture might be relatively easy to detect and support a fast and reliable strategy for categorization (cf. Cheetham et al., 2011). Alternatively, avatar-specifying information might relate to more detailed shape and texture information of the eyes, nose, and mouth (MacDorman et al., 2009a; Looser and Wheatley, 2010) independently of novelty processing. But it should be noted that visual discrimination performance of facial texture and shape properties is influenced by experience (Vuong et al., 2005; Balas and Nelson, 2010) and that perceptual and categorization experience can influence the selection of perceptual details used for analysis before categorization (e.g., Schyns, 1991; Schyns and Murphy, 1994). Given that novel stimuli are known to evoke different patterns of fixations than familiar stimuli (Althoff and Cohen, 1999), it is possible that repeated exposure to and greater perceptual and categorization experience with non-human faces might result in a change in eye movement patterns. Participants were therefore selected with a view to limiting the potential impact on eye movement behavior of active experience with building and modifying avatars in video games and virtual reality environments and of active gaming experience, while recognizing that active and incidental exposure to humanlike characters in other media (e.g., comics and books, movies, and commercials) could influence eye movement patterns. The influence on eye movement patterns of active and incidental perceptual and categorization learning with humanlike characters along the DHL awaits investigation.

Our main finding of the present study concerns the relative shift away from the nose region in avatars to the regions of the eyes and mouth of ambiguous faces. One approach to understanding which perceptual details are perceived and analyzed before categorization of these faces might be to consider the role of spatial frequency information. Eye movements are selective for spatial frequency information (e.g., Tavassoli et al., 2009) and the nose region is more diagnostic for processing stimulus information in coarser spatial frequency scales (Schyns et al., 2002). Coarser frequencies (i.e., low spatial frequencies) are suggested to facilitate encoding of shape and texture on the basis of the general luminance properties and shading of the face, corresponding therefore to a faster and broader but less detailed processing of the face (Sergent, 1986; Schyns and Oliva, 1994; Schyns and Gosselin, 2003). Given the relative shift in dwell time toward the nose region of avatars and the comparatively short RT for avatars, it is conceivable that a broad processing strategy would help the quick identification of perceptual information that indicates membership of the avatar category.

In contrast, longer response latencies and a greater proportion of dwell time to the eyes and mouth of ambiguous faces might be interpreted as reflecting a shift away from a faster but broad and less detailed processing strategy to a more time-consuming strategy of processing finer perceptual details (Schyns and Murphy, 1994; Lamberts, 1998; Johansen and Palmeri, 2002). This is especially likely for highly similar faces (e.g., Oliva and Schyns, 1997) and, presumably, for faces that are difficult to discriminate in terms of avatar or human category membership. The eye region is more diagnostic in finer frequency scales (Schyns et al., 2002) and the processing of the specific shape of the mouth and eyes and of the contours of the nose benefits from a finer spatial resolution (i.e., high spatial frequencies; Sergent, 1986; Schyns and Gosselin, 2003). We did not examine or manipulate spatial frequency, but these considerations suggest that there may have been a subtle shift in the relative salience of the nose of avatars to the eyes and mouth of ambiguous faces in a manner that resembles a *course-to-fine* sampling strategy along the DHL. This strategy would entail rapid and courser perceptual information processing that is sufficient for avatar categorization and more time-consuming processing of finer details (in addition to the coarser details) in order to disambiguate the category membership of faces near and at the category boundary. This *course-to-fine hypothesis* might be of further interest for uncanny-related research.

Women were faster than men to make category decisions. This is generally consistent with the reported female advantage in other face processing categorization tasks (e.g., Hall et al., 2010). But this finding applied only to ambiguous and human faces. In fact, the greatest difference between men and women in RT was at the category boundary. The category boundary is associated with enhanced perceptual discrimination ability along the DHL (Cheetham et al., 2011; Cheetham and Jancke, 2013), but these studies did not examine gender-related differences. Like the present study, these studies presented male faces only. Whether the findings of this study similarly apply for female stimuli awaits further investigation. Women do show a processing advantage for discriminating perceptual details in faces, especially under more demanding processing conditions (McBain et al., 2009; see also McGivern et al., 1998; Kimchi et al., 2008), but there were no gender-related differences in fixation or dwell time between avatar, human, and ambiguous faces, suggesting that any female advantage in RT does not translate into differences in eye movements along the DHL. The only gender-related difference in eye movements was in the general *face feature hierarchy*, with enhanced female attention to the eye region across the DHL for dwell time. A similar finding in women has been shown for expression recognition, and this was associated with greater eye-related dwell time and fixation number (Hall et al., 2010). Enhanced female attention to the eyes could be considered a task-independent effect associated with greater processing of socially relevant information in women (Saether et al., 2009).

In summary, these findings show that categorization ambiguity influenced the relative amount of dwell time spent extracting visual information from the eyes, nose, and mouth, though in comparison only with the non-human side of the DHL. Together with the shorter decision response latencies for avatar faces, the findings thus suggest that categorically unambiguous avatars are processed differently than ambiguous or human faces. Guided in part by current uncanny research, the focus of this first eye movement study of category decision making along the DHL was placed on the eyes, nose, and mouth. These internal face features were also selected because the greater part of eye sampling behavior was directed toward these regions in previous studies (e.g., Barton et al., 2006). But the arbitrary size and shape of the AOI used in the present study might well have masked potential findings. For example, our data show a consistent 70–75% of fixations and dwell time within the internal facial regions of all faces along the DHL, suggesting that the external face area is nevertheless relevant for avatar-human category decision making. External facial features such as the jaw line, hair profile, and head contour, which were masked in this study, might be informative in further studies of the DHL and category processing. We focused on highly realistic non-human faces, and our findings might apply for similarly highly realistic androids (see Saygin et al., 2012) but not necessarily for less humanlike avatars, cartoon faces, and robots (see Chen et al., 2010; Saygin et al., 2012). Our choice of stimuli did not reflect the examples of humanlike objects in the depiction of Mori’s hypothesis in Figure 1. This was because we sought to ensure careful experimental control and exclusion of visual cues of other perceptual dimensions that could confound with judgments of human likeness along the DHL (Cheetham and Jancke, 2013). We used 2D facial stimuli, but given the growing impact of 3D technology in various media, a promising avenue of further research would be to examine the effect of stereoscopic depth cues in 3D compared with 2D facial stimuli on eye sampling behavior and categorization (e.g., Bülthoff and Newell, 2000; Liu and Ward, 2006; Chelnokova and Laeng, 2011).

## Conflict of Interest Statement

The authors declare that the research was conducted in the absence of any commercial or financial relationships that could be construed as a potential conflict of interest.

## Footnotes

^1^http://www.smithmicro.com

^2^www.neurobs.com
